# Clusters of spatial, temporal, and space-time distribution of hemorrhagic fever with renal syndrome in Liaoning Province, Northeastern China

**DOI:** 10.1186/1471-2334-11-229

**Published:** 2011-08-26

**Authors:** Wei Wu, Junqiao Guo, Peng Guan, Yingwei Sun, Baosen Zhou

**Affiliations:** 1 Department of Epidemiology, School of Public Health, China Medical University, Shenyang, PR China; 2 Liaoning Provincial Center for Disease Control and Prevention, Shenyang, PR China

## Abstract

**Background:**

Hemorrhagic fever with renal syndrome (HFRS) is a rodent-borne disease caused by Hantavirus, with characteristics of fever, hemorrhage, kidney damage, and hypotension. HFRS is recognized as a notifiable public health problem in China, and Liaoning Province is one of the most seriously affected areas with the most cases in China. It is necessary to investigate the spatial, temporal, and space-time distribution of confirmed cases of HFRS in Liaoning Province, China for future research into risk factors.

**Methods:**

A cartogram map was constructed; spatial autocorrelation analysis and spatial, temporal, and space-time cluster analysis were conducted in Liaoning Province, China over the period 1988-2001.

**Results:**

When the number of permutation test was set to 999, Moran's I was 0.3854, and was significant at significance level of 0.001. Spatial cluster analysis identified one most likely cluster and four secondary likely clusters. Temporal cluster analysis identified 1998-2001 as the most likely cluster. Space-time cluster analysis identified one most likely cluster and two secondary likely clusters.

**Conclusions:**

Spatial, temporal, and space-time scan statistics may be useful in supervising the occurrence of HFRS in Liaoning Province, China. The result of this study can not only assist health departments to develop a better prevention strategy but also potentially increase the public health intervention's effectiveness.

## Background

Hemorrhagic fever with renal syndrome (HFRS) is a rodent-borne disease caused by Hantavirus, with characteristics of fever, hemorrhage, kidney damage, and hypotension [1-3]. HFRS is recognized as a notifiable public health problem in China [4]. Currently, HFRS is endemic in 28 of 31 provinces in mainland China [5]. HFRS is severely endemic in mainland China and accounts for 90% of the total cases reported worldwide [6], and remains a significant public health problem with 20 000-50 000 human cases diagnosed annually [7]. Liaoning Province is one of the most seriously affected areas with the most cases in China.

The occurrence of HFRS is regular in space, time and space-time. It is important to study the cluster patterns of HFRS to establish the risk factors behind the spread of HFRS, and to prevent and control HFRS better. Obviously, It is necessary to conduct scan statistics, which are an elegant way to solve problems of multiple testing when there are closely overlapping spatial areas and/or time intervals being evaluated. Scan statistics are now commonly used for disease cluster detection and evaluation, for many diseases including infectious diseases [8-10], cancer [11-14], cardiology [15], auto-immune diseases [16] and liver diseases [17]. The scan statistics software SaTScan [18] is usually used to perform the temporal, spatial, and space-time analyses.

A previous study has analyzed the spatial distribution of HFRS in Liaoning Province, China, during 2000 to 2005 using spatial scan statistics methods. A geographic area was identified in Eastern Liaoning Province as the most likely endemic cluster [19]. There are some differences between our study and theirs. First, we also conduct the temporal and space-time analyses besides spatial analyses. Second, the time period of our study was during 1988 to 2001. Third, Liaoning Province was divided into 100 study areas in our study, while in theirs was divided into 58 study areas.

This study examined the excess variation, and whether such excesses were temporary or stable. The objective of this study was to determine whether areas and periods with HFRS had homogeneous characteristics in Liaoning Province from 1988 to 2001. The department of public health can also use this information to assess the effectiveness of HFRS control.

## Methods

Liaoning Province is located in the southern part of China's Northeast. There are 19 counties, 8 autonomic counties, 17 county level cities, and 56 districts in Liaoning Province, therefore 100 study areas were included in our study. The information included the number of HFRS cases per year in every area from 1988 to 2001. For the 14-year period, the average annual incidence was 3.6 cases per 100 000 persons, and 20 227 cases were involved in this study. Records on HFRS cases between 1988 and 2001 were obtained from Liaoning Provincial Center for Disease Control and Prevention.

To conduct scan statistics on the spatial, temporal, and space-time distribution of HFRS, a polygon map of Liaoning Province at a scale of 1:1 000 000 was obtained, on which the point layers that contained information regarding latitudes and longitudes of central points of each area were created. Demographic information based on the Liaoning Province Statistical Yearbook was integrated in terms of an administration code. All HFRS cases were geo-coded and matched to the layers on the polygons and points by administration code using the software ArcGIS9.2.

To describe the upper outliers and lower outliers in different areas, a circular cartogram was produced. The size (area) of the circles is proportional to the value of the selected variable [20]. In this study the hinge used to identify outliers was set to be 1.5.

In spatial autocorrelation analysis, Moran's I spatial autocorrelation statistic and its visualization in the form of a Moran Scatter Plot were used to investigate autocorrelations. The number of permutation tests was set to 999 and the pseudo-significance level was set at 0.001.

Spatial clustering occurs if a disease event is seen more often in a particular area than would be expected by chance. The clusters of disease cases are meaningful only after adjustment for spatial variations in the density of the background population. The SaTScan methods have been widely used in spatial cluster analysis. SaTScan uses circles and a non-parametric test statistics [18]. In this study, retrospective spatial cluster analysis for higher incidence was used, in which the maximum spatial cluster size was set at < 50% of the total population at risk to find possible sub-clusters.

The space-time scan statistic is defined by a cylindrical window with a circular (or elliptic) geographic base and with height corresponding to time. In this study, retrospective space-time cluster analysis for higher incidence was used, in which the maximum spatial cluster size was set at 50% of the total population at risk, and the maximum temporal cluster size was set at 50% of the total population at risk to find possible sub-clusters.

The temporal scan statistic uses a window that moves in 1 dimension, time, defined in the same way as the height of the cylinder used by the space-time scan statistic. This means that it is flexible in start and end date. In this study, retrospective temporal cluster analysis for higher incidence was used, in which the maximum temporal cluster size was set at 50% of the total population at risk to find possible sub-clusters.

For each window, the method uses a Monte Carlo simulation to test the null hypothesis that there was not an elevated risk of HFRS. Under the Poisson assumption, the likelihood function for a specific window is proportional to:

$${\left(\frac{c}{E\left[c\right]}\right)}^{c}{\left(\frac{C-c}{C-E\left[c\right]}\right)}^{C-c}I\left(\right)$$

where C is the total number of cases, c is the observed number of cases within the window and E[c] is the covariate adjusted expected number of cases within the window under the null hypothesis. I() is an indicator function. When SaTScan is set to scan only for clusters with high rates, I() is equal to 1 when the window has more cases than expected under the null hypothesis. The window with the maximum likelihood is the most likely cluster, that is, the cluster least likely to be due to chance. For each window of movable position and size change, the software tested the risk of HFRS within and outside the window using the null hypothesis of the same risk.

## Results

There were a total of 20 227 HFRS cases reported in Liaoning Province, China, from 1988 to 2001. The annual average incidence ranged from 0.00 to 26.80 per 100 000.

**Figure 1:**
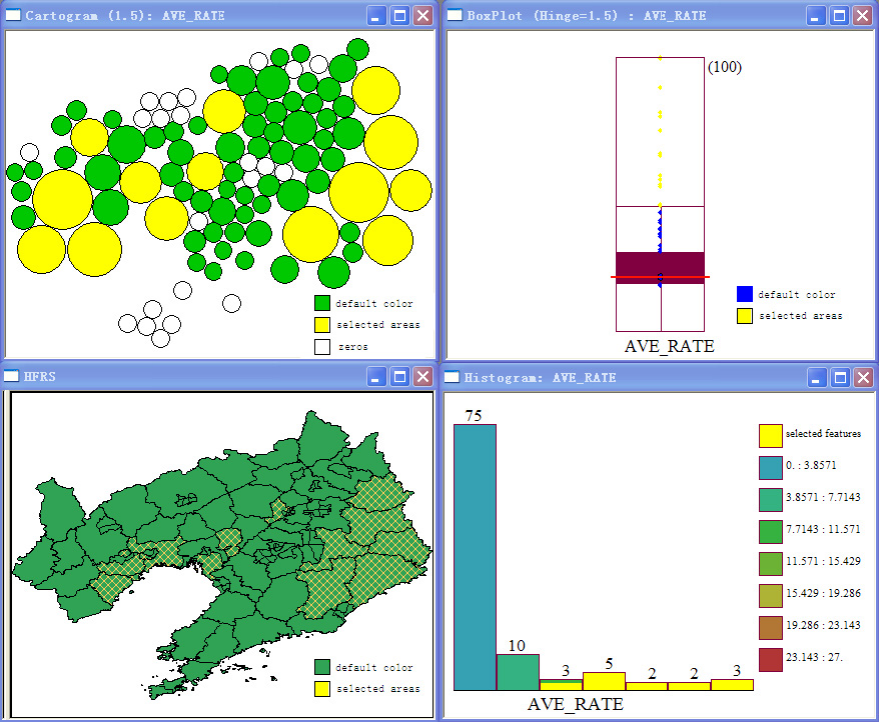
**Cartogram of haemorrhagic fever with renal syndrome in Liaoning Province, China, 1988-2001**.

Zero and outliers are shown in different size (area) and color in the circular cartogram. When the hinge used to identify outliers was set to be 1.5, 14 areas were recognized as upper outliers, and there were no lower outlier (Figure 1).

**Figure 2:**
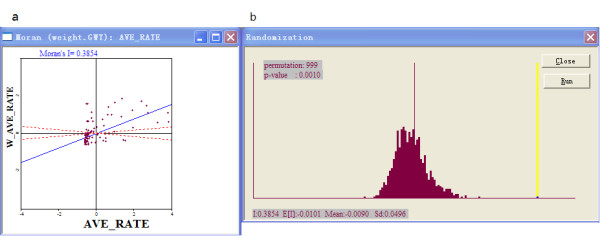
**Global spatial autocorrelation analysis for annual average incidence of HFRS in Liaoning Province, China from 1988 to 2001**. a. The Moran scatter plot for annual average incidence of HFRS. b. the histogram for significance assessment of Moran's I.

A Moran scatter plot was created and a significance assessment through a permutation test was implemented by global spatial autocorrelation analysis for annual average incidence of HFRS (Figure 2). The number listed on the top of the graph (0.3854) is the Moran's I statistic (Figure 2a). A histogram was created by performing the significance assessment of the Moran's I statistic (Figure 2b). In addition to the reference distribution (in brown) and the statistic (as a yellow bar), also listed in the graph were the number of permutations (999) and the pseudo-significance level (0.001) in the upper left corner, as well as the value of the statistic (0.3854), its expected mean (E [I] = -0.0101), and the mean and standard deviation of the empirical distribution were -0.0090 and 0.0496 respectively. The statistic turned out to be significant for Moran's I at significance level of 0.001. Spatial autocorrelation analysis for annual incidence of HFRS in Liaoning Province, China from 1988 to 2001 showed that the Moran's I statistic was significant from 1990 to 2001 at a significance level of 0.05 (Table 1), while it was not significant in 1988 and 1989.

**Table 1:** Spatial autocorrelation analysis for annual incidence of HFRS in Liaoning Province, China from 1988 to 2001.

Year	Moran's I	E(I)	Mean	SD	*P*-value
1988	0.0348	-0.0101	-0.0095	0.0435	0.1260
1989	0.0609	-0.0101	-0.0124	0.0415	0.0620
1990	0.0958	-0.0101	-0.0123	0.0450	0.0280
1991	0.1525	-0.0101	-0.0094	0.0433	0.0060
1992	0.1023	-0.0101	-0.0060	0.0485	0.0270
1993	0.1262	-0.0101	-0.0107	0.0366	0.0160
1994	0.2838	-0.0101	-0.0104	0.0479	0.0010
1995	0.2492	-0.0101	-0.0088	0.0476	0.0010
1996	0.2712	-0.0101	-0.0094	0.0474	0.0010
1997	0.3260	-0.0101	-0.0096	0.0508	0.0010
1998	0.2596	-0.0101	-0.0120	0.0462	0.0010
1999	0.3131	-0.0101	-0.0110	0.0411	0.0010
2000	0.3772	-0.0101	-0.0116	0.0452	0.0010
2001	0.3819	-0.0101	-0.0079	0.0485	0.0010

**Figure 3:**
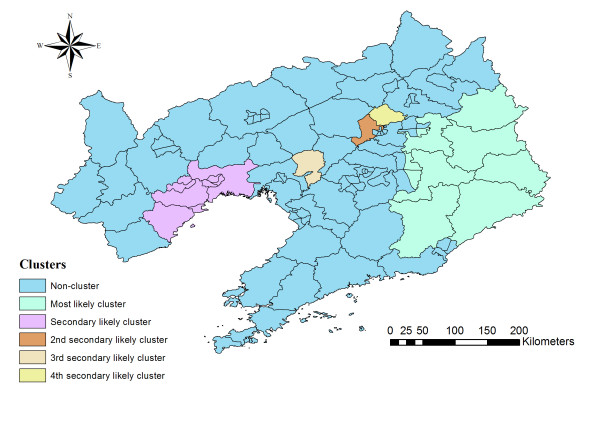
**Spatial distribution of identified clusters of HFRS cases with significant higher incidences in Liaoning Province, China from 1988 to 2001**.

Spatial cluster analysis of cases of HFRS in 1988-2001 in Liaoning Province showed HFRS was not distributed randomly in space. Using the maximum spatial cluster size of ≤50% of the total population, one most likely cluster and four secondary clusters were identified (Table 2 Figure 3). The overall relative risk (RR) within the most likely cluster was 5.679 (*P *= 0.001), with an observed number of cases of 6635 compared with a calculated 1600.94 expected cases. The RR of secondary clusters within a nonrandom distribution pattern was also significant (*P *= 0.001).

**Table 2:** SaTScan statistics for spatial clusters with significant higher incidence in Liaoning Province, China from 1988 to 2001.

Type	Location	Case	Expected	Relative risk	*P*-value
Most likely	Hengren county, Xinbin county, Kuandian county, Benxi county, Qingyuan county, Nanfen district, Fushun county, Xihu district, Wanghua district, Fengcheng city	6635	1600.94	5.679	0.001

Secondary	Longgang district, Lianshan district, Xingcheng city, Taihe district, Nanpiao district, Linghe district, Guta district, Linghai city	5934	1409.95	5.541	0.001

2^nd ^Secondary	Yuhong district	628	183.62	3.498	0.001

3^rd ^Secondary	Tai'an county	478	181.07	2.680	0.001

4^th ^Secondary	Xinchengzi district	279	150.64	1.864	0.001

**Figure 4:**
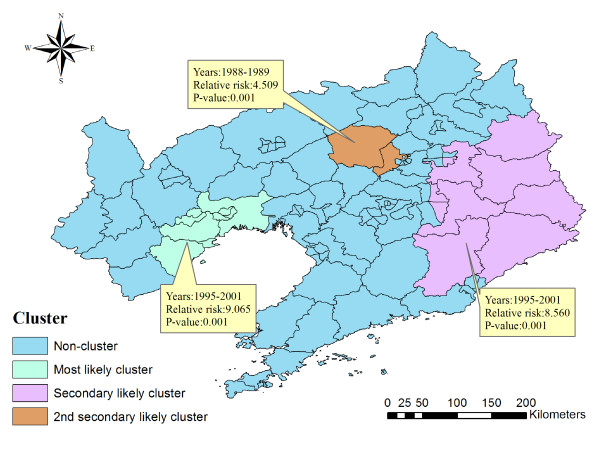
**Space-time distribution of identified clusters of HFRS cases with significant higher incidences in Liaoning Province, China from 1988 to 2001**.

Space-time cluster analysis of cases of HFRS in 1988-2001 in Liaoning Province showed that HFRS was not distributed randomly in space-time. Using the maximum spatial cluster size of 50% of the total population, and the maximum temporal cluster size of 50% of the total population, one most likely cluster and two secondary clusters were identified (Table 3 Figure 4). The overall RR within the most likely cluster was 9.065 (*P *= 0.001) with an observed number of cases of 5119 compared with a calculated 728.79 expected cases. The RR of secondary clusters within a non-random distribution pattern was also significant (*P *= 0.001).

**Table 3:** SaTScan statistics for space-time clusters with significantly higher incidence in Liaoning Province, China from 1988 to 2001.

Type	Location	Time frame	Case	Expected	Relative risk	*P*-value
Most likely	Longgang district, Lianshan district, Xingcheng city, Taihe district, Nanpiao district, Linghe district, Guta district, Linghai city	1995-2001	5119	728.79	9.065	0.001

Secondary	Hengren county, Xinbin county, Kuandian county, Benxi county, Qingyuan county, Nanfen district, Fushun county, Xihu district, Wanghua district, Fengcheng city	1995-2001	5279	801.41	8.560	0.001

2^nd ^Secondary	Xinmin city, Yuhong district	1988-1989	320	71.86	4.509	0.001

Temporal cluster analysis of cases of HFRS in 1988-2001 in Liaoning Province showed HFRS was not distributed randomly in time. Using the maximum temporal cluster size of 50% of the total population, one most likely cluster was identified (Table 4). The overall RR within the cluster was 3.195 (*P *= 0.001) with an observed number of cases of 11529 compared with a calculated 5931.51 expected cases, and there was no secondary cluster identified.

**Table 4:** SaTScan statistics for temporal clusters with significantly higher incidence in Liaoning Province, China from 1988 to 2001.

Type	Time frame	Case	Expected	Relative risk	*P*-value
Most likely	1998-2001	11529	5931.51	3.195	0.001

Secondary	-	-	-	-	-

## Discussion

Spatial, temporal and space-time scan statistics are new tools to detect aggregation of disease cases, to test the occurrence of any statistically significant clusters, and ultimately to find evidence of risk factors. Scan statistics also identify whether cases of disease in space or time can be explained by chance or are statistically significant. The spatial scan statistic method is a useful instrument for HFRS analysis of cluster patterns. The space-time scan statistic method is proposed as a dynamic supplement to purely spatial statistical methods for outbreak detection and prediction. Aiming prevention strategies at areas of highest risk can potentially increase the public health intervention's effectiveness. People at highest risk should be informed of the high risk and the possibilities for risk reduction. The result of this study will assist health departments to develop a better prevention strategy. Further epidemiological studies will be conducted in populations defined by the study to gain insights into specific risk factors.

We investigated the spatial distribution of HFRS cases and identified areas with high endemicity of HFRS and clustering patterns with spatial scan statistics. We showed that in the period from 1988 to 2001 as a whole, the geographic distribution patterns of HFRS cases in Liaoning Province were not random. Spatial cluster analysis identified one most likely cluster and four secondary clusters based on a maximum spatial cluster size of 50% of total population.

We identified the eastern areas as having one most likely cluster in Liaoning Province which was also reported by Hualiang Lin [19]. It is possible to think of Liaoning Province as three approximate geographical regions: the highlands in the west, plains in the middle, and hills in the east. The eastern part of Liaoning Province is dominated by the Changbai Shan and Qianshan ranges, which extends into the sea to form the Liaodong Peninsula. The most likely clusters of HFRS in Liaoning Province were mainly located in the hills. As for the secondary cluster, our result was different from that of Hualiang Lin's although with the same parameters. We found other three secondary clusters (Yuhong district, Tai'an county and Xinchengzi district) besides the western mountainous regions. The reasons could be as follows. First, the time we studied was different. The period was during 2000 to 2005 in the other study, whereas it was during 1988-2001 in our study. Second, the other study considered the city-governed region and others at a county level; therefore there were 58 county-level study areas, whereas in our study, Liaoning Province was divided into 100 study areas. In this study, we also conduct the temporal and space-time analyses besides spatial analyses.

After calculating the Moran's I statistic annually, it was significant from 1990 to 2001 at a significance level of 0.05, while it was not significant in 1988 and 1989. This implied that the distribution of HFRS in Liaoning Province from 1990 to 2001 was not distributed randomly in space, while in 1988 and 1989, it was distributed randomly. It is likely that environmental factors contribute to the epidemic and development of HFRS. Some studies have shown that some factors are related to the high incidence of HFRS such as environmental and climatic factors [21,22]. Clearly, these factors could also affect the geographical clustering. It is necessary for us to study the changes in these factors between 1988 and 1989 and 1990 and 2001 in a future study.

We also conducted a space-time cluster analysis besides the purely spatial cluster analysis. Using the maximum spatial cluster size of 50% of the total population, and the maximum temporal cluster size of 50% of the total population, we identified one most likely cluster and two secondary clusters. When we compared the most likely and secondary clusters of the purely spatial cluster analysis with those of the space-time cluster analysis, we found an interesting phenomenon. The areas of the most likely cluster in the spatial cluster analysis became the areas of the secondary cluster in the space-time cluster analysis. Vice versa, the areas of the secondary cluster in the spatial cluster analysis became the areas of the most likely cluster in the space-time cluster analysis. That is to say, if we regarded the period 1988-2001 as a whole, the 10 areas, Hengren County, Xinbin County, Kuandian County, Benxi County, Qingyuan County, Nanfen District, Fushun County, Xihu District, Wanghua District, Fengcheng City had a higher RR than the 8 areas, Longgang District, Lianshan District, Xingcheng City, Taihe District, Nanpiao District, Linghe District, Guta District, Linghai City. The RR was 5.679 and 5.541 respectively. However, during the period 1995-2001, the 8 areas had a higher RR than the 10 areas if we considered both space and time. The RR was 9.065 and 8.560 respectively. Therefore, space-time disease-surveillance methods should be proposed as a dynamic supplement to purely spatial statistical methods for outbreak detection to detect and predict localized outbreaks before they spread to larger regions.

Temporal cluster analysis identified one most likely cluster based on a maximum temporal cluster size of 50% of the total population which had a significantly (*P* < 0.001) increased HFRS risk, and the time frame was 1998-2001. In this analysis, we considered Liaoning Province as a whole place, and only considered the influence of time for HFRS.

## Conclusions

The present study analyzed the statistically significant spatial, temporal and space-time clusters of HFRS in Liaoning Province, China. The cluster analysis in this study was as an ecological study; therefore some weakness could not be avoided. The quality of data over time and from different places may be quite variable, and the ecological fallacy is usually interpreted as a major weakness. However, the cluster analysis does provide valuable information about geographic and/or temporal disparity of HFRS for further study in Liaoning Province, China. The result of this study can not only assist health departments to develop a better prevention strategy but also potentially increase the public health intervention's effectiveness. Future research could focus on more detailed individual level investigations. To implement specific risk-reduction programs, the use of such cluster analysis tools should become integral components in the epidemiological description and risk assessment of infectious diseases.

## Competing interests

The authors declare that they have no competing interests.

## Authors' contributions

BZ conceived the study and obtained the foundation. BZ and JG designed the study. WW and PG designed and performed the statistical analysis. YS prepared the manuscript. All authors contributed to subsequent revisions and approved the final version.

## Pre-publication history

The pre-publication history for this paper can be accessed here:

http://www.biomedcentral.com/1471-2334/11/229/prepub
